# Fluidized-Bed Granulation of Probiotics-Encapsulated Spray-Dried Skim Milk Powder: Effects of a Fluidizing Aid, Moisture-Activation and Dehydration

**DOI:** 10.3390/foods10071600

**Published:** 2021-07-09

**Authors:** Dong-Hyun Lim, Andres Letona, Minjeong Lee, Dayoung Lim, Nam-Soo Han, Donghwa Chung

**Affiliations:** 1 Food Technology Major, Graduate School of International Agricultural Technology, Seoul National University, Pyeongchang 25354, Korea; wladimich@naver.com (D.-H.L.); pffoahtm@snu.ac.kr (M.L.); dy.lim@snu.ac.kr (D.L.); 2 Institute of Food Industrialization, Institutes of Green Bio Science and Technology, Seoul National University, Pyeongchang 25354, Korea; nanobox8@snu.ac.kr; 3 Department of Food Science and Biotechnology, Chungbuk National University, Cheongju 28644, Korea; namsoo@cbnu.ac.kr; 4 Center for Food and Bioconvergence, Seoul National University, Seoul 08826, Korea

**Keywords:** fluidized-bed granulation, *Lactobacillus rhamnosus* GG, spray drying, moisture-activation, flowability

## Abstract

A probiotic powder of poor flowability with high dust content, prepared by spray drying reconstituted skim milk fermented with *Lactobacillus rhamnosus* GG (LGG), was granulated by fluidized-bed granulation (FBG). The effects of the addition of skim milk powder (SMP) as a fluidizing aid, and of simple moisture-activation with or without dehydration, were investigated with respect to the performance of the FBG process. A fine, poorly fluidizable LGG powder (Geldart Group C) could be fluidized and granulated, with a 4- to 5-fold increase in particle size (*d*_4,3_ = 96–141 μm), by mixing with SMP (30–50%), which has larger, fluidizable particles belonging to Geldart Group A. Moisture-activation after the mixing, followed by fluidized-bed dehydration with hot air to remove excess moisture, further improved the FBG; the yield of the granules increased from 42% to 61% and the particle size distribution became much narrower, although the average particle size remained almost the same (*d*_4,3_ = 142 μm). These granules showed a popcorn-type structure in scanning electron microscopy images and encapsulated a sufficient level of viable LGG cells (1.6 × 10^8^ CFU g^−1^). These granules also exhibited much better flowability and dispersibility than the spray-dried LGG powder.

## 1. Introduction

Spray drying often produces fine powders (<50 µm) that are difficult to handle, process, and store due to their poor flowability and reconstitution behavior, as well as their dust-like nature [1,2,3,4]. An effective way to minimize these issues is to enlarge the size of the powder particles using a four-step granulation process: wetting and nucleation, coalescence or growth, consolidation, and attrition or breakage [5]. Wet granulation, a process involving the displacement of air on the particle surface with binding liquid, is one of the most popular granulation techniques used in industry [3,4,5,6]. The two most popular wet granulation processes are high-shear wet granulation (HSWG) and fluidized-bed granulation (FBG). HSWG is strongly influenced by the mechanical redispersion of the binding liquid by impellers and choppers, whereas FBG is primarily influenced by the wetting of particles with binding liquid [7].

During FBG, fine particles exist in a fluid-like state due to the stream of inlet gas. The fine particles bind together via liquid bridges formed by the sprayed binding liquid, and the excess liquid is removed by simultaneous drying, resulting in the formation of large granules [8,9,10,11]. The granules obtained by FBG are porous and have a narrower particle size distribution than the fine particles before FBG.

The fluidization behavior of particles is the primary factor in the success of FBG. Geldart [12] provided a standard classification for predicting particle fluidization behavior based on the surface area-weighted mean diameter (*d*_3,2_) and particle true density (*ρ*_true_). According to this standard, particles are classified into four groups: A, B, C, and D (Supplementary Materials Table S1). Particles belonging to Group A (*d*_3,2_ = 30–100 µm, *ρ*_true_ < 1400 kg m^−3^) are aeratable and well fluidize, showing significant bed expansion without bubbling [11,12]. In contrast, particles belonging to Group C (*d*_3,2_ < 20 µm, *ρ*_true_ < 1400 kg m^−3^) show very poor fluidization behavior with significant formation of bubble channels [11,12]. These fine particles have strong interparticle cohesive forces and behave like clusters of particles, rather than independent particles, during fluidization. An effective approach to enhance the fluidization of Group C particles is to mix them with a fluidizing aid, such as well-fluidizing larger particles (e.g., Group A particles) [13,14]. Another promising way to improve the fluidization of fine particles is to perform pre-granulation, such as moisture-activated dry granulation, before FBG [15,16]. However, little information is currently available on the development of FBG process for fine, poorly fluidizable dairy powders belonging to Geldart Group C.

In this study, a fine probiotic powder (Geldart Group C particles) with poor flowability and high dust, prepared by directly spray drying the reconstituted skim milk (RSM) fermented with *Lactobacillus rhamnosus* GG (LGG), was granulated using FBG to improve the applicability of the probiotic powder. The effects of the addition of skim milk powder (SMP, Geldart Group A particles) as a fluidizing aid, and of moisture-activation with or without dehydration, were investigated with respect to the performance of the FBG process. Water was used as a binder, and hot-air fluidization was used for dehydration instead of using chemical moisture absorbent, to minimize the alteration of physicochemical properties of original powder and also possible adverse effect on probiotics activity.

## 2. Materials and Methods

### 2.1. Materials

LGG (ATCC 53103) was purchased from the American Type Culture Collection (Manassas, VA, USA). SMP (carbohydrate 52% *w*/*w*, sugars 49% *w*/*w*, protein 37% *w*/*w*, lipid 0.5% *w*/*w*, sodium 0.54% *w*/*w*, calcium 1% *w*/*w*), de Man, Rogosa, and Sharpe (MRS) broth, yeast extract, and glucose were obtained from Seoul Milk Co., Ltd. (Seoul, Korea), Difco Laboratories Inc. (Detroit, MI, USA), Thermo Fisher Scientific (Erembodegem, Belgium), and Ducksan Pure Chemicals Co., Ltd. (Asan, Korea), respectively.

### 2.2. Preparation of LGG-Fermented RSM

The LGG was subcultured sequentially for 24 and 18 h at 37 °C in 20 mL of MRS broth. The RSM medium (190 mL) was prepared with 10% (*w*/*w*) SMP, 2% (*w*/*w*) glucose, and 1% (*w*/*w*) yeast extract in distilled water and heat-treated at 90 °C for 30 min in a water bath. The subculture was inoculated (5%, *w*/*w*) into the heat-treated RSM medium, followed by incubation at 42 °C in a water bath with stirring at 100 rpm until the pH decreased from 6.6 to 3.9 (~9.2 log CFU g^−1^).

### 2.3. Spray Drying of LGG-Fermented RSM

The feed suspension for spray drying was prepared by adding SMP to the LGG-fermented RSM to a final concentration of 30% (*w*/*w*) with stirring for 30 min. Spray drying of the feed suspension was conducted using a laboratory-scale spray dryer (Eyela SD-1000; Tokyo Co. Ltd., Tokyo, Japan), having a single nozzle of 0.7 mm diameter and a current airflow system. Spray drying was conducted under the following conditions: inlet temperature = 160 ± 1 °C, outlet temperature = 80 ± 1 °C, feed flow rate = 300 mL h^−1^, atomization pressure = 100 kPa, and hot air flow rate = 0.20–0.24 m^3^ min^−1^. The resulting powder was cooled to room temperature in the dryer and designated as LRP.

### 2.4. Fluidized-Bed Granulation of Spray-Dried Powders

**Figure 1:**
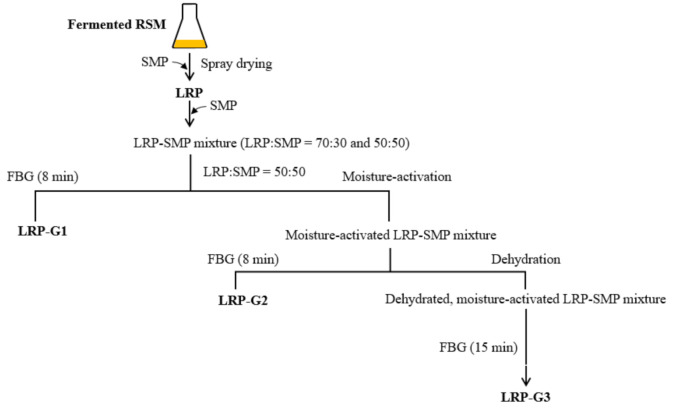
Experimental scheme. RSM, reconstituted skim milk; SMP, skim milk powder; FBG, fluidized bed granulation; LRP, LGG-fermented RSM powder; LRP-G1, granules obtained by FBG of LRP-SMP mixture (50:50); LRP-G2, granules obtained by FBG of moisture-activated LRP-SMP mixture (50:50); LRP-G3, granules obtained by FBG of moisture-activated, dehydrated LRP-SMP mixture (50:50).

FBG of LRP-based powders (Figure 1) was performed using a laboratory-scale fluidized-bed (3 L chamber volume; BD-600S; IREA Tech Co. Ltd., Daejeon, Korea). The powders (50 g) were fluidized at an inlet temperature of 50 °C with an air flow rate of 0.15 m^3^ min^−1^. Distilled water was used as a binding liquid and top-sprayed (0.7 mm nozzle diameter) at 100 kPa during the fluidization with a flow rate of 0.8 mL min^−1^.

The effects of a fluidizing aid on FBG were examined by mixing SMP, as a fluidizing aid, with LRP at an LRP:SMP ratio of 70:30 or 50:50 (*w*/*w*). The mixtures of LRP and SMP underwent FBG for 8 min under the conditions described above. The granules obtained by FBG of the LRP-SMP mixture (50:50) were designated as LRP-G1.

The effects of moisture-activation on FBG were investigated with and without dehydration. The LRP-SMP mixture (50:50) was sprayed with distilled water (2.5%, *w*/*w*) using a hand sprayer during a 5-min mixing process conducted at room temperature with an impeller speed of 500 rpm. This water-sprayed mixture was sieved through a 1-mm mesh screen to remove excessively large agglomerates, and then allowed to flow via FBG for 8 min under the conditions described above. The granules obtained by this process were designated as LRP-G2. Meanwhile, the sieved water-sprayed LRP-SMP mixture was further dehydrated for 20 min in the fluidized-bed system with an inlet temperature of 50 °C and air flow rate of 0.15 m^3^ min^−1^. During dehydration, the mixture was sampled (about 1 g) at 5-min intervals, and the changes in viable cells, moisture content, and water activity were measured to determine the proper dehydration time. The mixture dehydrated for 5 min was granulated by FBG for 8 or 15 min under the conditions described above. The granules obtained with 15-min FBG were designated as LRP-G3.

### 2.5. Microstructure and Particle Size Analyses

The particle microstructure was examined using scanning electron microscopy (SEM; Tabletop Microscope TM3030 Plus; Hitachi, Tokyo, Japan). The micrograph (1000×) was acquired at a voltage of 5 kV. The volume-weighted mean diameter (*d*_4,3_, µm) of particles, and the particle size distribution, were determined using a laser diffraction particle size analyzer (1190LD; CILAS, Orleans, France). The number, size, and volume of peaks in the particle size distribution were analyzed. The surface area weighted-mean diameter (*d*_3,2_, µm) was also determined for Geldart classification of LRP and SMP.

### 2.6. Density Measurement

The particles were equilibrated at 25 °C and zero *a*_w_ in a desiccator saturated with phosphate pentoxide before measuring the density. The true density (*ρ*_true_, kg m^−3^) of SMP and LRP was measured using a gas pycnometer (Ultrapyc 1200e; Quantachrome Instruments, Boynton Beach, FL, USA), and used together with *d*_3,2_ for Geldart classification of the particles. The loose bulk density (*ρ*_lb_, kg m^−3^) and tapped bulk density (*ρ*_tb_, kg m^−3^) were determined for SMP, LRP, and LRP-G3. The particles (10 g) equilibrated were loaded into a 50-mL graduated cylinder. The *ρ*_lb_ values were then determined based on the particle volume without tapping. The cylinder was tapped up and down manually until the particle volume no longer changed, and the *ρ*_tb_ values were obtained based on the particle volume after tapping [17,18].

### 2.7. Moisture Content and Water Activity Measurements

The moisture content (*X*, % dry basis) of the particles was determined by drying at 105 °C for 24 h in a drying oven [19]. The water activity (*a*_w_) of the particles was determined at 25 °C using a digital water activity meter (Series 3 TE; Aqualab, Decagon, WA, USA).

### 2.8. Determination of Flowability

The Carr compressibility index (CI) and Hausner ratio (HR) were calculated using the following equations, with *ρ*_lb_ and *ρ*_tb_ values measured as described above [17,18]. The flowability of particles was determined according to the criteria in Table S2 [20].

(1) $$\mathrm{CI}=\frac{\left(\rho_{\mathrm{tb}}-\rho_{\mathrm{lb}}\right)}{\rho_{\mathrm{tb}}}\times 100$$

(2) $$\mathrm{HR}=\frac{\rho_{\mathrm{tb}}}{\rho_{\mathrm{lb}}}$$

### 2.9. Dispersibility Measurement

The dispersibility of LRP, SMP, and LRP-G3 was determined according to Balde and Aïder [21] and Schuck et al. [22], with slight modifications. The particles were equilibrated at 25 °C and zero *a*_w_ in a desiccator saturated with phosphate pentoxide. One gram of each particle was poured into a 50-mL beaker containing 10 mL of distilled water at 20 °C, followed by vigorous shaking (25 times for 15 s). The dispersion was filtered through a 200-μm sieve, and 1 mL of the filtrate was taken and dried at 105 °C for 24 h in a drying oven to obtain a dry solid mass. The dispersibility (%) was calculated using the following equation:

(3) $$\mathrm{Dispersibility}=\frac{\left(p+w\right)\times \mathrm{TS}}{p\left(\frac{100}{100+X}\right)}$$

where *p* is the amount of particles used (g), *w* is the amount of water used (g), TS is the total solid content of the filtrate (%, $\mathrm{TS}\;=\frac{\mathrm{dry}\;\mathrm{solid}\;\mathrm{mass}\;\mathrm{of}\;\mathrm{filtrate}}{\mathrm{total}\;\mathrm{mass}\;\mathrm{of}\;\mathrm{filtrate}}\times 100$), and *X* is the moisture content of the particles (% dry-basis). A dairy powder having a dispersibility higher than 95% is considered dispersible [22].

### 2.10. Survivability Measurement

The particles (1 g) were dispersed in 9 mL of sterile saline solution (0.85% NaCl) and decimally diluted. Aliquots of the dilutions were spread on the sterile MRS agar plates and incubated at 37 °C for 48 h. The viable cell count was expressed as log CFU g^−1^. The survivability (%) of LGG after the formation of LRP-G3 was calculated using the following equation [23].

(4) $$\mathrm{Survivability}=\frac{\left(\mathrm{viable}\;\mathrm{cell}\;\mathrm{count}\;\mathrm{of}\;\mathrm{LRP}-G3\right)\times 2}{\mathrm{viable}\;\mathrm{cell}\;\mathrm{count}\;\mathrm{of}\;\mathrm{LRP}\;}\times 100$$

### 2.11. Statistical Analysis

All experiments were conducted at least in triplicate. Data were expressed as mean ± standard deviation. Statistical differences between data were determined by Student’s t-test, performed using SigmaPlot (version 10.0; Systat Software, Inc., San Jose, CA, USA), at a confidence level of 0.05.

## 3. Results and Discussion

### 3.1. Geldart Classification of Spray-Dried Probiotic Powder

Our preliminary experiments indicated that the LRP (spray-dried RSM-based powder encapsulating LGG) was poorly fluidized and not suitable for FBG. On the other hand, SMP showed good fluidizing behavior (data not shown). The LRP had an *X* value of 5.68%, *d*_3,2_ value of 9 µm, and *ρ*_true_ value of 1330 kg m^−3^. According to the Geldart classification (Table S1) [11,12,13,14], the LRP is classified as a Group C powder, specifically, a fine, very poorly fluidized powder with significant channel formation, which shows particle-cluster behavior rather than individual particle behavior. Compared to the LRP, SMP (*X* = 3.95%) was much larger (*d*_3,2_ = 35 µm) and had a significantly lower density (*ρ*_true_ = 1180 kg m^−3^); as such, it is classified as a Group A powder, showing aeratable and good fluidizing behavior. In this study, therefore, SMP was mixed as a fluidizing aid to improve the flowability of LRP and, consequently, the performance of FBG.

### 3.2. Effect of SMP on Fluidized-Bed Granulation of LRP

**Figure 2:**
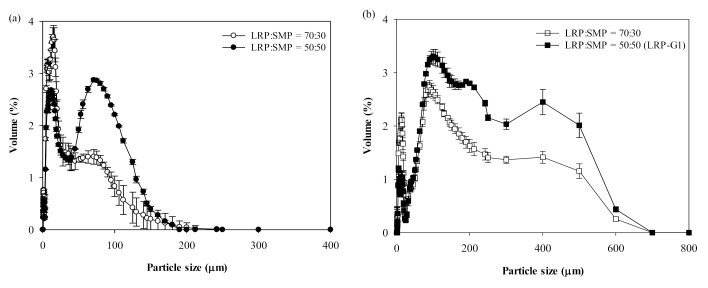
Particle size distribution of (**a**) LRP-SMP mixtures and (**b**) their granules obtained by fluidized-bed granulation. The granules obtained from the LRP-SMP mixture (50:50) were denoted as LRP-G1.

Figure 2a shows the particle size distribution obtained after the addition of SMP to LRP at different ratios (30% or 50% SMP). Three major peaks were observed for both LRP-SMP mixtures: Peak 1 (*d*_4,3_ ≈ 2–7 µm), Peak 2 (*d*_4,3_ ≈ 27 µm), and Peak 3 (*d*_4,3_ ≈ 80–112 µm) (Figure 2a; Table S3). The mixture of 30% SMP showed peak volume fractions of about 25%, 60%, and 5% for Peaks 1–3, respectively; however, the volume fraction of Peak 1 decreased to about 7%, and that of Peak 3 increased to about 41%, when the fraction of SMP in the mixture increased to 50% (Table S3).

Figure 2b shows the particle size distribution of the granules prepared by FBG of the two LRP-SMP mixtures, in which the two types of granules also showed three major peaks. The granules of 30% SMP had *d*_4,3_ values of about 2, 18 and 371 µm for Peaks 1–3, respectively, in which Peaks 2 and 3 were predominant (36 and 60% peak volume fractions, respectively; Table S3). The granules of 50% SMP (LRP-G1) showed much larger *d*_4,3_ values for the three peaks (about 16, 209, and 488 µm, respectively, for Peaks 1–3), in which Peak 2 was the major peak (77% volume fraction). The overall *d*_4,3_ value increased from 19 to 96 µm (about 5-fold), and from 34 to 141 µm (about 4-fold), after FBG of the LRP-SMP mixtures of 30% and 50% SMP, respectively (Table S3). Taken together, these results indicate that the fine LRP, which was very poorly fluidized, could be fluidized and granulated by FBG when SMP was used as a fluidizing aid, and the average particle size increased by about 4- to 5-fold after FBG. The addition of SMP (larger, lower-moisture particles belonging to Geldart Group A) could significantly reduce the strong interparticle cohesive forces of LRP by decreasing particle surface area [11,12] and surface moisture, thus allowing smooth particle fluidization. Once fluidized, the particles underwent agglomeration by sprayed water. Both LRP and SMP consist of carbohydrates, proteins, lipids, and minerals. The carbohydrates on particle surface might undergo glass transition due to the plasticizing effect of sprayed water, and thus the particle surface became sticky when the surface viscosity became lower than 10^8^ Pa s, forming either water-bridges or sinter-necks between particles [24,25]. Although the lipid content in LRP and SMP is small (<1% *w*/*w*), lipid is mostly located on the outer layer of particle surface and thus can melt during FBG and cause particle agglomeration [24]. Despite the particle enlargement by FBG, the granules obtained still had a broad particle size distribution (span = 3.49–5.17; Table S3). In addition, the yield of granules was only about 42%, due to the particle loss caused mainly by the elutriation and entrainment of fine particles during FBG [26,27,28]. To improve the performance of FBG, moisture-activation (with or without successive dehydration) was adopted prior to FBG.

### 3.3. Effect of Moisture-Activation (without Dehydration) on Fluidized-Bed Granulation of LRP-SMP Mixture

**Figure 3:**
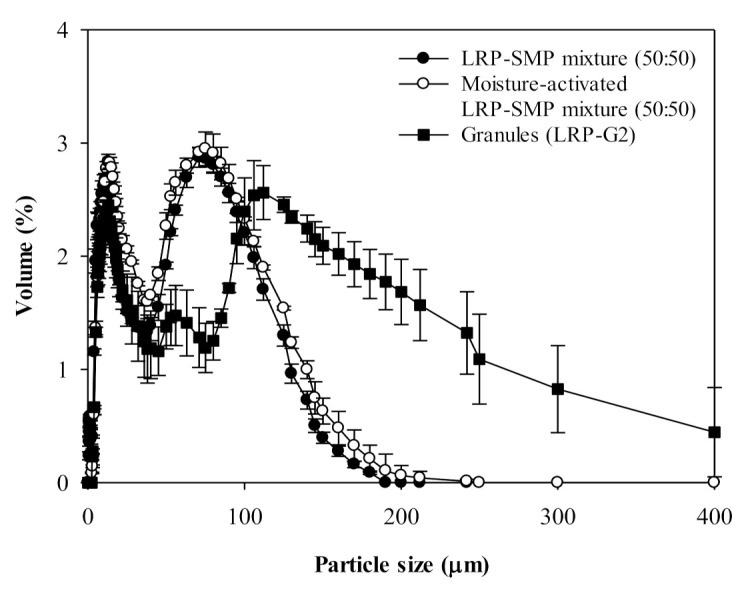
Particle size distribution of LRP-SMP mixture (50:50), moisture-activated LRP-SMP mixture, and the granules obtained by fluidized-bed granulation of moisture-activated LRP-SMP mixture. These granules were denoted as LRP-G2.

Figure 3 shows the particle size distribution of LRP-SMP mixture (50:50) before and after moisture-activation by water-spraying (2.5%, *w*/*w*), and the granules (LRP-G2) obtained by FBG of the moisture-activated LRP-SMP mixture. Moisture-activation is the process of activating particle agglomeration by facilitating the formation of either water-bridges or sinter-necks between particles using a small amount of water [14,15,16,29]. The small fraction of the finest particles in the LRP-SMP mixture (Peak 1) disappeared after moisture-activation, indicating consolidation of the finest particles into larger agglomerates due to the formation of either water-bridges or sinter-necks. However, the other two peaks remained almost unchanged in size and volume fraction (Table S4). The smaller particles tended to form water-bridged or sinter-necked agglomerates more easily than the larger particles, due to their higher affinity to water resulting from a higher surface area-to-volume ratio [30].

The granules (LRP-G2) showed three major peaks in their size distribution; Peak 1 (*d*_4,3_ ≈ 33 µm), Peak 2 (*d*_4,3_ ≈ 65 µm), and Peak 3 (*d*_4,3_ ≈ 290 µm) (Figure 3; Table S4). The particles of Peaks 1 and 3 accounted for most of the particle volume (51 and 41% volume fractions, respectively; Table S4). Compared to LRP-G1, LRP-G2 had a larger fraction of small particles and did not have big (about 500 µm) particles. This is probably because the moisture-activation created more regions of excess moisture, and the particles in this region were partially dissolved and dried without proper formation of water-bridges or sinter-necks between particles during the FBG. Consequently, the overall *d*_4,3_ of LRP-G2 (about 75 µm) was 2.2-fold smaller than that of LRP-G1 (141 µm; Table S3). The span of LRP-G2 (6.57) was also larger than that of LRP-G1 (3.49; Table S3). The results indicate that the moisture-activation may not be effective for improving the FBG performance of the LRP-SMP mixture. As in the general MADG process, the dehydration step may be required after moisture-activation to enhance the efficiency of FBG by removing excess water from the particles [31].

### 3.4. Effect of Dehydration on the Properties of Moisture-Activated LRP-SMP Mixture

Dehydration of the moisture-activated LRP-SMP mixture (50:50) was performed using the fluidized-bed system with hot air (50 °C), instead of using a moisture absorbent such as microcrystalline cellulose (MCC) or colloidal silicon dioxide, to simplify the granulation process and the mixing of other substances, as well as to minimize the alteration of physicochemical properties of original powder and possible adverse effect of moisture absorbent on probiotics activity.

**Figure 4:**
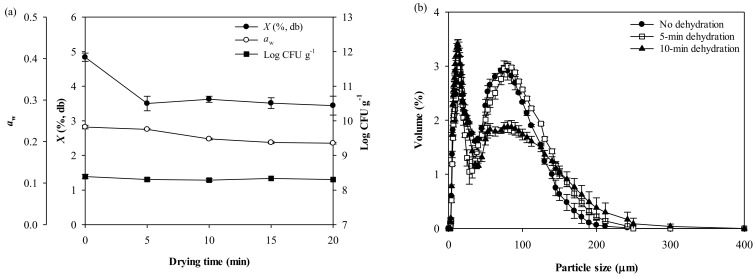
(**a**) Changes in the viable cells, moisture content (*X*), and water activity (*a*_w_) of moisture-activated LRP-SMP mixture (50:50) during 20-min dehydration at 50 °C, and (**b**) the particle size distribution of the mixture treated with 5- or 10-min dehydration or without dehydration.

Figure 4a shows that the moisture content (*X*) of the moisture-activated LRP-SMP mixture decreased sharply, from 4.8 to 3.5%, during the initial 5-min dehydration and remained almost unchanged during the next 15-min dehydration. The water activity (*a*_w_) also decreased from 0.24 to 0.20 due to the dehydration. The viable cells remained unchanged (about 2.0 × 10^8^ CFU g^−1^) during the entire 20-min dehydration process. This may be due to the protective effects of heat-denatured proteins and calcium ions in RSM [32]. Additionally, the LGG cells may gain more heat-resistance, due to their pre-exposure to heat stress during spray drying [33,34].

Figure 4b shows the particle size distribution of the moisture-activated LRP-SMP mixture treated with 5- or 10-min dehydration. The two major size peaks of the mixture were still observed after dehydration. The 5-min dehydration increased the *d*_4,3_ and volume fraction of Peak 2 (larger size particle group), from 120 to 132 µm and from 43% to 50%, respectively, resulting in a slight increase in the overall *d*_4,3_ from 39 to 43 µm (Table S5). The 10-min dehydration resulted in a sharp increase in the *d*_4,3_ of Peak 2 to 151 µm; however, the volume fraction of Peak 2 decreased significantly to 36%, which is a much smaller value than that of the 5-min dehydrated mixture (50%). For this reason, the 10-min dehydrated mixture showed a smaller overall *d*_4,3_ value (36 µm) than the 5-min dehydrated mixture (Table S5). The 10-min dehydration produced larger agglomerated particles; however, these particles had more collisions with other particles and the wall due to the longer duration in the fluidized-bed system, resulting in more breakage (or attrition) of particles. Considering these results, the 5-min dehydrated mixture was further granulated by FBG.

### 3.5. Effect of Moisture-Activation (with Dehydration) on Fluidized-Bed Granulation of LRP-SMP Mixture

**Figure 5:**
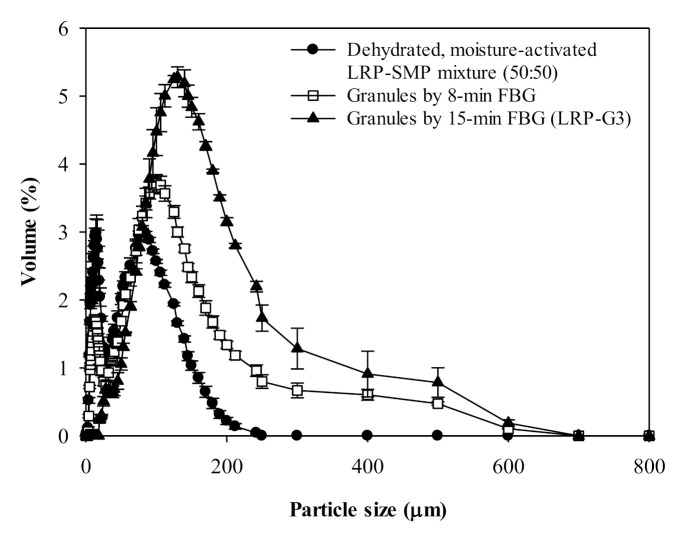
Particle size distribution of moisture-activated LRP-SMP mixture (50:50) treated with 5-min dehydration and the granules obtained by 8- or 15-min fluidized-bed granulation of the mixture. The granules from 15-min process were denoted as LRP-G3.

Figure 5 shows the particle size distribution of the moisture-activated LRP-SMP mixture treated with 5-min dehydration before and after FBG. The two major size peaks were still observed after FBG. However, the *d*_4,3_ of Peak 2 (larger size particle group) increased significantly from 132 to 302–310 µm after FBG, and the volume fraction of Peak 2 reached 97% after the 15-min FBG (Table S6). The overall *d*_4,3_ increased 3.3-fold (from 43 to 142 µm) after the 15-min FBG, and the span was greatly reduced to 1.68. The 15-min FBG (LRP-G3, *X* = 5.05%) showed a higher yield (61%) than the LRP-G1 (42%). The results indicate that the LRP (Geldart Group C powder) was successfully granulated with greatly reduced fine particle content by the following process; mixing with SMP (50%, Geldart Group A powder), 5-min moisture-activation, 5-min dehydration, and 15-min FBG.

**Figure 6:**
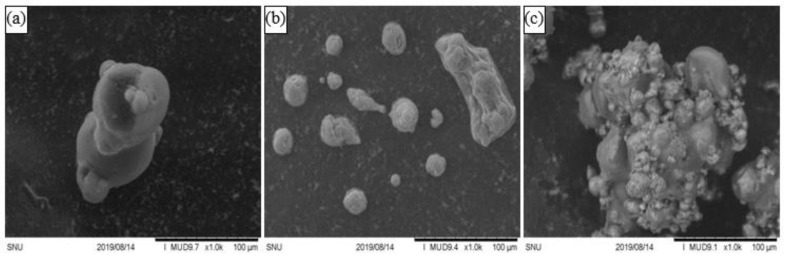
SEM images of (**a**) SMP, (**b**) LRP, and (**c**) LRP-G3.

SEM revealed that the LRP-G3 granules had a popcorn-type structure, in which agglomeration of small particles into granules was clearly visible (Figure 6). This structure is typically observed for granules obtained by FBG [6,7]. Table 1 shows that when the LRP was granulated to LRP-G3, the loose bulk density (*ρ*_lb_) and tabbed bulk density (*ρ*_tb_) decreased significantly, from 430 to 330 kg m^−3^ and from 750 to 460 kg m^−3^, respectively, due to the increase in particle size. The CI and HR values of LRP were 42.9% and 1.75, respectively (Table 1), indicating that the LRP has extremely poor flowability according to the criteria in Table S2 [20]. On the other hand, the LRP-G3 granules showed a significant reduction in CI and HR (27.4% and 1.38, respectively; Table 1), indicating that the flowability of LRP was greatly improved by FBG. The increase in particle size via FBG decreased the particle surface area, resulting in a reduction in friction between the particles, and in the cohesion of particles induced by van der Waals attractions between particles [35]. However, it should be noted that the flowability of LRP-G3 was still classified as poor according to the criteria in Table S2. The dispersibility of LRP was only 49.7%, while LRP-G3 showed a greatly increased dispersibility of 91.6%, slightly smaller than the value of SMP (95.6%) (Table 1). The reconstitution of food powders in water occurs via several steps: the wetting of particles by water, penetration of water by capillary rise, immersion of particles into water, and dissolution of particles in water, which often occur simultaneously and influence each other [36]. The significant increase in dispersibility after granulating LRP to LRP-G3 can be attributed primarily to the large increase in particle diameter, which can enlarge the capillary diameter between particles, thus promoting water penetration into particle packages according to Washburn’s capillary rise theory [36]. It should be noted that the dispersibility of LRG-G3 is slightly below 95%, above which a dairy powder is considered to be dispersible [22]. During FBG of LRP to LRP-G3, the amount of viable LGG cells only decreased slightly, from 4.1 × 10^8^ to 1.6 × 10^8^ CFU g^−1^. Taking into account the mixing of LRP with SMP (50:50), the survivability (Equation (4)) of LGG cells during FBG of LRP to LRP-G3 was calculated to be 80.2%. The lactose contained in SMP in a high amount (~49% *w*/*w*) is known to be an effective cell protecting agent, which can replace water molecules hydrating on polar groups of membrane phospholipids and proteins, and thus reduce damage to bacterial membranes and proteins [37,38]. Not only the stress via FBG, but also particle entrainment, may be responsible for this small loss of LGG cells during the process [39].

## 4. Conclusions

This study demonstrated that LRP, consisting of fine and very poorly fluidizable particles (Geldart Group C), was successfully granulated (LRP-G1) when fluidized together with SMP, which consists of larger, lighter, and well-fluidizable Geldart Group A particles. The LRP-G1 had an approximately 4–5-fold larger particle size than LRP, but still showed a broad particle size distribution (span = 3.49) and a production yield of less than 50% (42%). To improve FBG, the mixture of LRP and SMP was moisture-activated prior to FBG, which eliminated the small fraction of the finest particles in the LRP-SMP mixture via consolidation of the finest particles into larger agglomerates. However, the resulting granules (LRP-G2) showed a smaller particle size and larger span value than LRP-G1, probably because the excess moisture region was largely formed in the LRP-SMP mixture due to moisture-activation, and the particles in this region were partially dissolved and did not properly form water-bridges or sinter-necks between particles during FBG. Fluidized-bed dehydration for 5 min with hot-air after moisture-activation greatly improved the performance of FBG, without significant loss of viable cells. Finally, the LRP-G3 was obtained using a granulation process consisting of the following steps: mixing LRP with SMP (50%), 5-min moisture-activation, 5-min dehydration, and 15-min FBG. The LRP-G3 had an average particle size (*d*_4,3_ = 142 µm) similar to LRP-G1 but showed a much-improved particle size distribution and an increased yield (61%). The LRP-G3 had a popcorn-type structure with a moisture content of 5.05% and sufficient level of viable LGG cells (1.6 × 10^8^ CFU g^−1^) and exhibited markedly enhanced flowability and dispersibility relative to LRP. The present study proposes a simple and effective FBG process consisting of SMP addition, moisture-activation, dehydration, and fluidized-bed agglomeration for a poorly fluidizable probiotic powder obtained by spray drying LGG-fermented RSM. This process can be easily applied for FBG of other food powders that are difficult to fluidize.

## Figures and Tables

**Table 1:** Loose bulk density (*ρ*_lb_), tapped bulk density (*ρ*_tb_), Carr Index (CI), Hausner ratio (HR), and dispersibility of LRP, LRP-G3, and SMP ^1^.

Particles ^2^	*ρ*_lb_ (kg m^−3^)	*ρ*_tb_ (kg m^−3^)	CI	HR	Dispersibility (%)
LRP	430 ± 0 ^b^	750 ± 0 ^a^	42.9 ± 0.01 ^a^	1.75 ± 0.00 ^a^	49.7 ± 5.9 ^c^
LRP-G3	330 ± 0 ^c^	460 ± 20 ^c^	27.4 ± 2.28 ^b^	1.38 ± 0.04 ^b^	91.6 ± 2.0 ^b^
SMP	500 ± 10 ^a^	640 ± 10 ^b^	21.2 ± 0.64 ^c^	1.27 ± 0.01 ^c^	95.6 ± 1.4 ^a^

^1^ Values with different letters in the same column are significantly different at *p* < 0.05 according to Student’s *t*-test. ^2^ LRP, LGG-fermented RSM powder; RSM, reconstituted skim milk; SMP, skim milk powder; LRP-G3, granules obtained by the following steps: mixing LRP with SMP (50%), 5-min moisture-activation, 5-min dehydration, and 15-min fluidized-bed granulation.

## Supplementary Materials

The following are available online at https://www.mdpi.com/article/10.3390/foods10071600/s1, Table S1: Geldart classification for particle fluidization [1]. Table S2: Classification of powder flowability according to Car compressibility index (CI) and Hausner ratio (HR) [20]. Table S3: Particle size (*d*_4,3_) and volume fraction of peaks in particle size distribution of LRP-SMP mixtures and their granules obtained by fluidized-bed granulation. The granules obtained from the LRP:SMP mixture (50:50) were denoted as LRP-G1. Table S4: Particle size (*d*_4,3_) and volume fraction of peaks in particle size distribution of LRP-SMP mixture (50:50), moisture-activated LRP-SMP mixture, and the granules obtained by fluidized-bed granulation of moisture-activated LRP-SMP mixture. The granules were denoted as LRP-G2. Table S5: Particle size (*d*_4,3_) and volume fraction of peaks in particle size distribution of the moisture-activated LRP-SMP mixture (50:50) treated with 5-min or 10-min dehydration at 50 °C or without dehydration.; Table S6: Particle size (*d*_4,3_) and volume fraction of peaks in particle size distribution of the moisture-activated LRP-SMP mixture (50:50) treated with 5-min dehydration before and after fluidized-bed granulation for 8 min or 15 min. The granules from 15-min process were denoted as LRP-G3.
