# Supplementary material for: Fluidized-Bed Granulation of Probiotics-Encapsulated Spray-Dried Skim Milk Powder: Effects of a Fluidizing Aid, Moisture-Activation and Dehydration
Source: Foods. 2021 Jul 9;10(7):1600. doi: 10.3390/foods10071600 (PMC8304935; doi:10.3390/foods10071600)
Supplement: Supplementary file 1 [file foods-10-01600-s001.zip › foods-1262414-supplementary.pdf]

Table S1

Geldart classification for particle fluidization [1].

Group	Fluidizing behavior	$d_{3,2}$ (μm)	ρ (kg m^{-3})
A (aeratable)	Aeratable and well-fluidizing	30–100	< 1,400
B (sand-like)	No smooth fluidization, bubble formation at the onset of fluidization	40–500	1,400–4,000
C (fine)	Very poor fluidization, significant formation of channels	< 20	< 1,400
D (large)	Largest particles fall in this group, requiring a large amount of gas during the fluidization	> 1,400	> 1,400

Table S2

Classification of powder flowability according to Car compressibility index (CI) and Hausner ratio (HR) [20].

CI (%)	Flow character	HR
$\leq$ 10	Excellent	1.00–1.11
11–15	Good	1.12–1.18
16–20	Fair	1.19–1.25
21–25	Passable	1.26–1.34
26–31	Poor	1.35–1.45
32–37	Very poor	1.46–1.59
>38	Very, very poor	>1.60

Table S3

Particle size ($d_{4,3}$) and volume fraction of peaks in particle size distribution of LRP-SMP mixtures and their granules obtained by fluidized-bed granulation. The granules obtained from the LRP:SMP mixture (50:50) were denoted as LRP-G1.

		LRP : SMP (w/w)			
		70:30		50:50	
		Mixture	Granule	Mixture	Granule (LRP-G1)
$d_{4,3}$ (μm) (volume fraction, %)	Peak 1	7.01 ± 0.01 (24.73 ± 0.34)	2.36 ± 0.99 (4.24 ± 0.09)	1.76 ± 0.01 (6.73 ± 0.17)	16.01 ± 0.22 (18.39 ± 0.44)
	Peak 2	26.60 ± 0.56 (60.07 ± 0.69)	18.36 ± 0.26 (35.99 ± 0.46)	27.68 ± 0.26 (52.41 ± 1.05)	209.03 ± 1.79 (76.71 ± 0.95)
	Peak 3	79.92 ± 2.31 (5.19 ± 1.00)	370.65 ± 5.62 (59.78 ± 0.38)	111.74 ± 1.25 (40.86 ± 1.22)	487.50 ± 0.51 (4.90 ± 0.51)
	Overall	18.75 ± 0.64	95.54 ± 4.11	33.70 ± 0.96	140.50 ± 3.54
Span		4.25 ± 0.15	5.17 ± 0.21	5.55 ± 0.17	3.49 ± 0.09

Table S4

Particle size ($d_{4,3}$) and volume fraction of peaks in particle size distribution of LRP-SMP mixture (50:50), moisture-activated LRP-SMP mixture, and the granules obtained by fluidized-bed granulation of moisture-activated LRP-SMP mixture. The granules were denoted as LRP-G2**.**

		LRP-SMP mixture	Moisture-activated LRP-SMP mixture	Granules (LRP-G2)
$d_{4,3}$ (μm) (volume fraction, %)	Peak 1	1.76 ± 0.01 (6.73 ± 0.17)	–	33.16 ± 0.62 (50.89 ± 2.16)
	Peak 2	27.68 ± 0.26 (52.41 ± 1.05)	28.29 ± 0.11 (56.70 ± 1.21)	64.91 ± 1.04 (7.66 ± 0.69)
	Peak 3	111.74 ± 1.25 (40.86 ± 1.22)	119.86 ± 8.53 (43.30 ± 1.21)	289.87 ± 62.79 (41.45 ± 1.47)
	Overall	33.70 ± 0.96	38.68 ± 1.46	74.67 ± 1.35
Span		5.55 ± 0.17	3.89 ± 0.06	6.57 ± 0.33

Table S5

Particle size ($d_{4,3}$) and volume fraction of peaks in particle size distribution of the moisture-activated LRP-SMP mixture (50:50) treated with 5-min or 10-min dehydration at 50 ^{o}C or without dehydration.

		No dehydration	5-min dehydration	10-min dehydration
$d_{4,3}$ (μm) (volume fraction, %)	Peak 1	28.29 ± 0.11 (56.70 ± 1.21)	19.25 ± 0.35 (49.57 ± 0.38)	26.62 ± 0.26 (64.38 ± 1.33)
	Peak 2	119.86 ± 8.53 (43.30 ± 1.21)	132.31 ± 3.08 (50.43 ± 0.38)	151.24 ± 14.23 (35.62 ± 1.33)
	Overall	38.68 ± 1.46	42.88 ± 0.48	36.25 ± 2.09
Span		3.89 ± 0.06	4.47 ± 0.07	5.87 ± 0.27

Table S6

Particle size ($d_{4,3}$) and volume fraction of peaks in particle size distribution of the moisture-activated LRP-SMP mixture (50:50) treated with 5-min dehydration before and after fluidized-bed granulation for 8 min or 15 min. The granules from 15-min process were denoted as LRP-G3.

		Before granulation	8-min granulation	15-min granulation (LRP-G3)
$d_{4,3}$ (μm) (volume fraction, %)	Peak 1	19.25 ± 0.35 (49.57 ± 0.38)	19.59 ± 0.28 (28.29 ± 0.61)	31.06 ± 0.26 (3.26 ± 0.25)
	Peak 2	132.31 ± 3.08 (50.43 ± 0.38)	310.02 ± 4.41 (71.71 ± 0.61)	301.94 ± 19.60 (96.74 ± 0.25)
	Overall	42.88 ± 0.48	85.82 ± 1.64	141.67 ± 7.41
Span		4.47 ± 0.07	2.67 ± 0.10	1.68 ± 0.18
